# Community Mobilization and Awareness Creation for Orofacial Cleft Services: A Survey of Nigerian Cleft Service Providers

**DOI:** 10.1155/2014/140713

**Published:** 2014-09-24

**Authors:** Raphael A. Adebola, Babatunde O. Bamgbose, Joshua B. Adeoye

**Affiliations:** ^1^Oral and Maxillofacial Surgery Department, Aminu Kano Teaching Hospital, Kano, Nigeria; ^2^Preventive Dentistry Department, Faculty of Dentistry, Bayero University, Kano, Nigeria; ^3^Oral Diagnostic Sciences Department, Faculty of Dentistry, Bayero University, Kano, Nigeria; ^4^Public Dental Health Department, Aminu Kano Teaching Hospital, Kano, Nigeria

## Abstract

*Background*. The opportunity to provide free surgical care for orofacial clefts has opened a new vista and is enhanced by well-informed communities who are aware of the free surgical services available to them. It is the responsibility of cleft care providers to adequately inform these communities via a combination of community mobilization and awareness creation. *Methods*. This was a nationwide, cross-sectional descriptive study of all orofacial cleft service providers in Nigeria using a structured, self-administered questionnaire. *Results*. A total of 4648 clefts have been repaired, 50.8% by the ten government-owned and 49.2% by the five nongovernment-owned organizations included in the study. The nongovernment-owned institutions seemed to be more aggressive about community mobilization and awareness creation than government-owned ones, and this was reflected in their patient turnout. Most of the organizations studied would prefer a separate, independent body to handle their awareness campaign. *Conclusion*. Community mobilization requires skill and dedication and may require formal training or dedicated budgets by government-owned and nongovernment-owned institutions alike. Organizations involved in cleft care provision must take community mobilization and awareness seriously if the largely unmet needs of orofacial cleft patients in Nigeria are to be tackled.

## 1. Introduction

Orofacial clefts (OFC) are a common congenital malformation of the head and neck and the prevalence in Nigeria is 0.5 per 1000 live births [1]. The incidence rate of cleft lip, cleft palate, and cleft lip and palate in blacks ranges from 0.18 to 1.67 per 1,000 [2]. Affected children often have difficulty feeding and require multidisciplinary medical and surgical care from birth to adulthood [3].

The impact of OFC is manifold, affecting the patients themselves and their immediate family on one hand and the society they belong to on the other [4, 5]. It affects physical functions such as speech, biting, sucking, and swallowing [6] and has diverse psychological effects on both the patient and their parents [4]. These psychological effects lead to different reactions, some as drastic as infanticide [7–9].

Different attempts have been made to quantify the psychological effects of the unaesthetic appearance of the cleft child and the impact of cleft lip repair on aesthetics [10–13]. Cleft lip repair is undertaken as early as 3 months of age, once the child is able to withstand the trauma of surgery, so as to reduce its psychosocial trauma on the parents [14]. This repair, though undertaken by surgeons, necessitates multidisciplinary intervention and includes the inputs of orthodontists, speech therapists, pediatricians, and counselors [7, 15].

The health and well-being of the patients are dependent upon the clinical expertise of those who serve them. In addition, society as a whole is affected by the quality of their care because the potential of the affected individual for a positive contribution to the community is inevitably influenced by the adequacy of treatment. There is a responsibility amongst cleft service givers to increase awareness about the availability, timing, advantages, and modalities of repair to counter the mostly negative attitudes of individuals and societies towards such a deformity [7].

Nigeria, the most populous country in Africa and the most populous black nation in the world with a population of over 160 million people [16] and a crude birth rate of 44 per 1000 [17], has an estimated prevalence rate of 0.5 per 1000 [1]. This estimated average of 3,360 OFC births per year combines with a significant number of unrepaired adult patients to present a huge disease burden. Awareness campaigns are imperative if all these clefts are to be repaired and if the psychosocial burden on cleft patients is to be alleviated.

The database of Smile Train, a US-based Nongovernmental Organization, NGO, that partners with OFC service providers to conduct free cleft surgeries all over Nigeria, has, however, recorded less than 7,000 free surgeries done in the country since inception more than ten years ago [18]. This low figure indicates a lack of awareness by patient and public of the availability of free treatment. This is despite the far spread of institutions known to perform cleft surgeries whether in consonance with or independent of the Smile Train.

Some private NGOs and privately owned hospitals and dental clinics also offer cleft lip repair services. It would seem that awareness about the availability of repair services available at these institutions, as well as others spread around these zones, is not enough, necessitating efforts at raising awareness and community mobilization.

“Awareness-raising” is a means of alerting specific groups and the public in general to the existence of OFC and the need to address it. It is a two-way street, fostering communication and information exchange in order to improve mutual understanding, whilst mobilizing communities and the wider society to bring about the necessary change in attitudes and behavior [19].

Community mobilization, on the other hand, is a capacity-building process through which community members, groups, or organizations plan carry out and evaluate activities on a participatory and sustained basis to improve their health and other conditions either on their own initiative or stimulated by others [20].

The opportunity to provide free surgical care for OFC has opened a new vista for research and treatment outcomes in Nigeria and can only be enhanced by well-informed communities who are aware of the challenges faced by these babies and their parents and the pivotal role of OFC repair in mitigating them.

The aim of this study is to survey the OFC service providers in Nigeria and assess the effectiveness of their community mobilization and awareness processes.

## 2. Materials and Methods

This was a nationwide cross-sectional descriptive study undertaken on all known orofacial cleft service providers in all geopolitical zones of Nigeria, including federally funded teaching hospitals and medical centers, nongovernmental organizations, and private dental clinics. Three (3) NGOs, 2 private dental clinics, and 10 federal government-owned orofacial cleft centers were included in the study.

Federal government-funded organizations were categorized as “government-owned organizations (GOOs)”, while private dental clinics and NGOs were grouped as “nongovernment owned organizations (NGOOs)”.

A structured self-administered questionnaire (the appendix) elicited information regarding methods of creating awareness and mobilizing target communities that were employed by the OFC services studied. The questionnaire also extracted information regarding financial and organizational commitments towards community mobilization and awareness-raising.

A formal IRB approval was not available for this study. However, the principles outlined in the Declaration of Helsinki were followed.

All statistical analyses were performed using Microsoft Excel for Mac and SPSS for Mac (version 18.0 SPSS Inc., Chicago, IL). Descriptive statistics were used and results expressed as frequencies and percentages in tables and charts.

## 3. Results

Fifteen (15) centers distributed across the 6 geopolitical zones of Nigeria were included in the study, and majority (60%) was located in the Northern region of the country. Of the fifteen, 10 were federal and 5 were privately owned institutions, including 3 NGOs and 2 dental clinics.

A total of 4648 cleft had been repaired, 50.8% by the 10 government-owned organizations (GOOs) and 49.2% by the 5 nongovernment owned organizations (NGOOs).  Table 1 presents the demographic and other features of the OFC service providers located in the six regions of the country.

Table 2 compares these institutions based on criteria related to community mobilization. Six (6) of the 10 GOOs did not have community mobilization groups as compared to 4 of the 5 NGOOs that did. Also, only 1 of the 10 provided formative assessment of the community perception of cleft lip and palate as compared to 2 of the 5 NGOOs that did.

All of the organizations provided cleft anomaly educational information to cleft care receivers (Table 3). Seven (7) of the 10 GOOs, however, did not provide training on feeding of cleft lip and palate patients and 9 of the 10 did not have formal training on advocacy skills as part of the community awareness training. These and other parameters related to community awareness are depicted in Table 3.

Access to the community is obtained through the gatekeepers and community arenas. Majority of the NGOOs combined multiple gatekeepers while gaining access to the community (Table 4), as compared to the GOOs that gained access predominantly through Primary Healthcare Centers or Local Government area Chairmen (Table 4).

Media employed by all organizations to create community awareness included print, electronic, and other more traditional media. Both sets of institutions seemed to prefer print to electronic or other traditional types of media, although the NGOOs employed a greater variety of media than the GOOs (Table 5).

Most subjects thought the most effective media were radio and posters (Table 6) while 11 of the 15 organizations studied (7 GOOs and 4 NGOOs) indicated their preference for a separate body, distinct from the surgical service, to handle awareness campaigns (Table 7).

## 4. Discussion

Community mobilization engages all sectors of the population in a community-wide effort to address a health, social, or environmental challenge. It brings together policy makers and opinion leaders, local, state, and federal government, professional groups, religious groups, businesses, and individual community members. It empowers individuals and groups to take some kind of action to facilitate change [21].

To our knowledge, no prior study has been done on community mobilization and awareness creation involving the Smile Train free cleft repair services. In our study, the NGOOs were more likely to engage in community mobilization than GOOs. One possible reason for this is the fact that the government hospitals are referral, tertiary-care centers and would not, ordinarily, need to mobilize the community to access their services. This is not the case with the NGOOs that may need to create community awareness for their services to have the desired impact.

A community is not merely a collection of individuals but a system that transcends those individuals. As a system it has various dimensions: technological, economic, political, institutional, ideological, and perceptual. Community participation does not happen by itself. It must be stimulated, encouraged, and facilitated [22].

Participation of communities is an essential element of community mobilization, but it is important to recognize that all participation is not equal. As community participation increases, community ownership and capacity increase, with the result that community action and continuous improvement in the quality of community life are more likely to be sustained over time [20]. Every community has opinion leaders, the people who make things happen in the community by virtue of their roles or positions. These people are the gatekeepers and should not be bypassed if community mobilization is to be effective [23]. They should, instead, be respectfully seen and engaged as agents of change. These opinion leaders are Kings, Chiefs/Emirs, Traditional Leaders, Religious Leaders, Political Leaders, Women Leaders, Youth leaders, and so forth [23]. In this study, NGOOs were more aggressive in engaging all types of gatekeepers in the community.

To mobilize the community for any particular intervention, a baseline understanding of its perception is necessary [24]. To do an effective community mobilization, a lot must therefore be known about the nature of communities in general and this information can be obtained through formal or informal research into the target community [22]. Our study shows that formative assessment is undertaken by very few of the organizations involved in cleft care in Nigeria. In generating awareness, it is important to make formative assessment of the community's concept of cleft lip and palate. Once communities understand the causes and consequences of clefts, they will be supportive of efforts at surgical repair.

In order to boost community mobilization for orofacial cleft services in a low socioeconomic environment like Nigeria, it is essential that certain steps (information dissemination, awareness raising, motivation, community mobilization, and total awareness) are prudently followed, to achieve the desired goals. Community mobilization, at its best, does not merely raise community awareness about cleft lip services; rather, it is a comprehensive strategy that includes the community action cycle [23]. The primary ingredients of a successful community mobilization program, termed “success factors,” consist of trained staff; adequate budget for media/publicity, transportation, and training; and educational materials.

The employment of most of these ingredients may explain why the 5 NGOOs studied repaired almost as many clefts as the 10 GOOs (Table 1). The 10 federal government-owned cleft centers had operated 2360 (50.8%), while the 5 nongovernment owned organizations had performed 2288 surgeries (49.2%) as per their records. This is more remarkable when the relative numbers of years these institutions had been into cleft care are taken into consideration (Table 1).

This may also however be linked to the fact that OFC repair is the priority service provided by some of the NGOOs studied, whereas cleft repair is a minor part of the activities in the government institutions. Furthermore, official bureaucracy in government institutions causes a lot of delay in access to care whereas such delays are not encountered at the NGOOs.

There is a statistically significant relationship between the level of educational attainment and the knowledge about cleft deformities [7]. This finding buttresses the need to adapt the medium and content of cleft deformity campaigns to the perceived level of education of the community. To create awareness among the less literate members of the society, the communication medium should be visual, and to make the message acceptable and reliable, a variety of media are required [22]. Most NGOOs in our study employed a variety of media, none more so than the Grassroot Smile Initiative (Table 1), an NGO, which employed all the mediums listed and who, unsurprisingly, posted the highest number of cleft surgeries.

The Owotade study showed that the most preponderant source of information about cleft deformities was family members [7]. In our study, we described that source of information as “word of mouth,” and it was not employed prominently by most of the institutions studied. The study instead showed a preference for radio and posters by both NGOOs and GOOs. Radios are affordable and most stations broadcast in local language and so, community members have closeness to this device. Posters are visual devices that can pass messages across at a glance and can be placed in multiple prominent places at once, cutting across all socioeconomic, educational, and locational divides.

It is also the experience of the authors that motor parks and drivers stationed therein were a veritable method of awareness creation. Commercial drivers travel across communities and are able to spread the news of cleft care through handbills, fliers, and word of mouth. They have frequently brought patients from distant locations to benefit from cleft care services.

Community mobilization also involves advocacy [22]. “Advocacy” is a set of actions, big or small, that aims to influence people who hold power to create positive change [22]. A successful advocacy campaign comprises a variety of actions that are deliberately planned to be complementary and build on each other. It should convince people that the desired social change is positive and beneficial. Advocacy has to be carefully planned to achieve clear and concise goals and objectives. The goals must, in turn, be specific, measurable, achievable, realistic, and time-bound. It requires research, planning, acting, and monitoring [22].

Advocacy is necessary to raise the awareness level of communities to cleft lip and palate services. People need to know about the availability of free cleft clinics and it would be important to identify the most effective ways of informing and sensitizing them about such services. This study showed that less than 30% of cleft service providers had any formal training in advocacy skills (Table 3). For a cleft repair service to grow, building community understanding is a challenge that must be addressed head on by the service providers.

The primary goal of awareness is fostering improved mutual understanding and mobilization of communities in such a way as to direct traffic to available cleft services [23]. “Awareness” is a two-way process where communities realize and understand that clefts can be repaired and know the appropriate action to take to benefit from such services [23]. “Awareness raising” is a multiway communication or interaction process which provides opportunities for dialogue, mutual learning, and trust-building, helping to empower communities and strengthen their interest [19].

Enhanced public awareness enables better-informed community participation and sustainable appreciation and patronage of the cleft services [19]. The objective of community sensitization and awareness strategies is to ensure the community actively participates in the cleft care available to them. This helps communities become key stakeholders in the cleft care provided by the organizations.

The methods of sensitization and awareness-raising include organizing workshops with communities; experience sharing from success stories; community participation and involvement; engaging the mass media and other institutions; using printed materials; involving popular personalities; implementing school programs; and meeting gatekeepers. Our study portrayed the sensitization methods utilized by both the NGOOs and the GOOs. The NGOOs focused more on awareness raising and community sensitization than the GOOs.

As indicated earlier, the about 7,000 free surgeries done in the country since inception of the Smile Train more than ten years ago are less than the estimated disease burden when prevalence and incidence values are taken into consideration [1, 16–18].

Possible explanations for the low number of treated orofacial clefts in Nigeria are poverty; patient's inability to afford transportation cost to treatment centers; fear of surgery; and misconception that other parts of the body may be cut to repair the cleft defect. Religious beliefs indicating that attempts to repair the defect amounted to questioning God's work have also been observed during interaction with some patients and patient relatives. Yet some others see the defect as a source of livelihood (as the children are used for alms begging).

In order for the number of orofacial cleft repairs done by both sets of institutions to approach the estimated disease burden, (the study could only account for 4646 in Table 1) the discussed ingredients of community mobilization and awareness-creation have to be employed more strategically and aggressively by the government and nongovernment owned institutions alike.

The government institutions may also need to have dedicated cleft units that make cleft repairs a priority service and empower such units with the authority to control their activities. Also, there may be a need to establish awareness-creation and community mobilization subunits in these institutions to increase their impact in their locales.

When asked if they would prefer to have an independent organization handle community mobilization, 11 of the 15 organizations (7 GOOs and 4 NGOOs) said “yes” to a separate body handling awareness campaign. This is an indication of the workload involved in awareness campaign and the difficulty of superimposing such demands on an organization that is more designed to delivering surgical care. A dedicated budget and independent awareness group will undoubtedly increase the efficiency of the cleft care organization in both government-owned and nongovernment owned institutions alike.

## 5. Conclusion

The Nongovernment owned organizations appear to be more aggressive in community mobilization and awareness-creation for free cleft surgical services, and this has reflected in the patient turnout, when compared with government-owned organizations, which, generally have been involved in the provision of cleft care for longer periods.

Community mobilization requires skill and dedication, which necessitate formal training and make it essential for organizations to have dedicated programs and, if possible, budgets in order to be able to fully mobilize the community. Many cleft service organizations would also prefer an independent group handle their awareness campaign.

## Figures and Tables

**Table 1:** Detailed characteristics of cleft care centers.

Geographic location [*n* = 15] (%)	Name of institution	Type of institution	Duration of existence (years)	Clefts repaired (%) [*n* = 4648]
North-West 3 (20%)	Ahmadu Bello University Teaching Hospital (ABUTH), Zaria	Teaching hospital	>10 years	72 (1.55)
	Aminu Kano Teaching Hospital (AKTH), Kano	Teaching hospital	5–10 years	155 (3.33)
	Grassroot Smile Initiative (GSI), Kano	Nongovernmental organization	<5 years	1246 (26.8)

North-Central 4 (26.7%)	Cleft Care Foundation	Nongovernmental organization	<5 years	300 (6.45)
	OHAI	Nongovernmental organization	<5 years	540 (11.6)
	Jos University Teaching Hospital (JUTH), Jos	Teaching hospital	5–10 years	129 (2.78)
	Vic Memorial Hospital, Jos	Private clinic	<5 years	52 (1.12)

North-East 2 (13.3%)	Federal Medical Center (FMC), Gombe	Medical center	5–10 years	380 (8.17)
	Metro Cons Clinic, Gombe	Private clinic	<5 years	150 (3.23)

South-West 3 (20.0%)	Lagos University Teaching Hospital (LUTH), Lagos	Teaching hospital	5–10 years	420 (9.04)
	Obafemi Awolowo University Teaching Hospital (OAUTH), Ile-Ife	Teaching hospital	>10 years	200 (4.30)
	University College Hospital (UCH), Ibadan	Teaching hospital	5–10 years	200 (4.30)

South-East 1 (6.7%)	National Orthopedic Hospital (NOH), Enugu	Federal specialist hospital	>10 years	454 (9.76)

South-South 2 (13.3%)	University of Benin Teaching Hospital (UBTH), Benin	Teaching hospital	>10 years	250 (5.38)
	University of Port-Harcourt Teaching Hospital (UPTH), Port-Harcourt	Teaching hospital	>10 years	100 (2.15)

Federal government-owned cleft center: 8 teaching hospitals, 1 specialist hospital, 1 medical center; total number of surgeries 2360 (50.8%).

Nongovernment owned organizations; 3 NGOs, 2 private dental clinics; total number of surgeries 2288 (49.2%).

**Table 2:** Community mobilization activities.

Activities	Type of institution	Yes	No
Mobilization/awareness group	**Both **	**8**	**7**
	Government-owned	4	6
	Nongovernment owned	4	1

Formative assessment of community perception of cleft lip and palate	**Both**	**3**	**12**
	Government-owned	1	9
	Nongovernment owned	2	3

Meeting with community gatekeepers	**Both **	**6**	**9**
	Government-owned	2	8
	Nongovernment owned	4	1

Budget for awareness campaign	**Both **	**5 **	**10 **
	Government-owned	2	8
	Nongovernment owned	3	2

Nutritional program for malnourished cleft lip and palate babies	**Both **	**6**	**9**
	Government-owned	3	7
	Nongovernment owned	3	2

Transportation of patients and parents	**Both**	**9**	**6**
	Government-owned	5	5
	Nongovernment owned	4	1

**Table 3:** Community awareness training and activities.

Training and activities	Type of institution	Yes	No
Cleft anomaly educational information to cleft care receivers	**Both **	**15**	**0**
	Government-owned	10	0
	Nongovernment owned	5	0

Training of caregivers on feeding of cleft lip and palate patients	**Both **	**6**	**9**
	Government-owned	3	7
	Nongovernment owned	3	2

Early identification of children with cleft lip and palate	**Both**	**6**	**9**
	Government-owned	4	6
	Nongovernment owned	2	3

Formal training on advocacy skills	**Both **	**4**	**11**
	Government-owned	1	9
	Nongovernment owned	3	2

**Table 4:** Access to the community.

Who/where visited	Type of institution	Yes	No
Traditional rulers	**Both **	**6**	**9**
	Government-owned	2	8
	Nongovernment owned	4	1

Local government area chairmen	**Both **	**6**	**9 **
	Government-owned	3	7
	Nongovernment owned	3	2

Religious leaders	**Both **	**5**	**10 **
	Government-owned	2	8
	Nongovernment owned	3	2

Primary health care centers	**Both **	**5**	**10 **
	Government-owned	3	7
	Nongovernment owned	2	3

Major markets	**Both **	**5 **	**10 **
	Government-owned	2	8
	Nongovernment owned	3	2

Motor parks	**Both **	**3**	**12 **
	Government-owned	0	10
	Nongovernment owned	3	2

Traditional birth attendants	**Both **	**1 **	**14 **
	Government-owned	0	10
	Nongovernment owned	1	4

Schools	**Both**	**3 **	**12 **
	Government-owned	1	9
	Nongovernment owned	2	3

**Table 5:** Awareness creation media.

Type of media	Type of institution	Yes	No
*Print media *			
Newspaper	**Both **	**3 **	**12 **
	Government-owned	1	9
	Nongovernment owned	2	3
Handbills	**Both **	**12**	**3 **
	Government-owned	7	3
	Nongovernment owned	5	0
Banners	**Both **	**6 **	**9 **
	Government-owned	3	7
	Nongovernment owned	3	2
Posters	**Both **	**11 **	**4 **
	Government-owned	6	4
	Nongovernment owned	5	0
*Electronic media *			
Radio jingles	**Both **	**8**	**7 **
	Government-owned	5	5
	Nongovernment owned	3	2
Television adverts	**Both **	**5**	**10 **
	Government-owned	3	7
	Nongovernment owned	2	3
Telephonic short message services (SMS)	**Both **	**2**	**13**
	Government-owned	0	10
	Nongovernment owned	2	3
*Other media *			
Town criers	**Both **	**2**	**13 **
	Government-owned	0	10
	Nongovernment owned	2	3
Souvenirs	**Both **	**3**	**12**
	Government-owned	2	8
	Nongovernment owned	1	4
“Word of mouth”	**Both **	**2**	**13 **
	Government-owned	1	9
	Nongovernment owned	1	4

**Table 6:** Most effective awareness media.

Type of media	Frequency of respondents
Radio	5
Posters	5
Souvenirs	1
No response	4

**Table 7:** Recommended separate “Awareness Organization.”

“Awareness Organization”	Frequency of respondents [*n*]
Institutional Information Unit	3
Independent Nongovernmental Organization	4
State Government Information Service or Ministry of Health	0
Federal Information Service or Ministry of Health	0
Combination of above	1
No suggestions	3

11 out of 15 organizations (7 governmental and 4 nongovernmental) said “Yes” to a separate body handling awareness campaign.

## Appendix

### Community Mobilization and Awareness Creation for Cleft Lip and Palate Services

Dear Sir/Madam

This questionnaire was designed to evaluate community mobilization and awareness creation for cleft lip and palate services in cleft treatment centers in Nigeria. Please respond appropriately to the following questions.

Thank you and God bless you.

Dr. Adebola R. A (Dean Faculty of Dentistry Bayero University Kano)

(1) Name of center———————————————
(2) Nature of centre (Tick as appropriate)
  (i) Teaching hospital
  (ii) Federal medical center
  (iii) State general hospital
  (iv) Private: NGO or individual practice
(3) How long has your center been into cleft care?
  (i) <5 years
  (ii) 5–10 years
  (iii) >10 years
(4) How many cleft patients have been treated since the inception of your team?
——————————————————————
(5) Does your team have a mobilization/awareness team?
  Yes
  No
(6) Has your awareness team carry out a formative assessment to understand the community context on cleft lip and palate?
  Yes
  No
(7) Has any formal meeting been organized with the community gatekeepers in your area on cleft care and availability of services by your team?
  Yes
  No
(8) Do you provide educational information about cleft anomalies to parents and patients, other professional people, and the general public?
  Yes
  No
(9) Do you provide educational programmes for hospital personnel and primary care providers addressing feeding and other critical aspects of early health care for children with cleft lip and palate?
  Yes
  No
(10) Do you promote early identification of children with cleft lip and palate through programmes designed to inform delivery room personnel, traditional birth attendants, and primary care providers in the community?
  Yes
  No
(11) Has your team been formally trained on advocacy skills on how to increase awareness on cleft lip and palate?
  Yes
  No
(12) Does your team have a budget for the awareness and mobilization programme?
  Yes
  No
(13) If yes, how much is budgeted monthly for the awareness programme?
(14) What percentage of your budget is allocated to the following:
  (a) Training
  (b) Transportation
  (c) Development of awareness materials
  (d) Media
  (e) Another direct cost associated with office expenses
(15) Indicate how your awareness message is delivered
  (a) Radio advert
  (b) Posters
  (c) Banners
  (d) Hand bills
  (e) Souvenirs
  (f) Television advert
  (g) Newsprints
  (h) Town criers
  (i) Telephone SMS
  (j) Others, please specify
(16) Has your team visited any of the following? Please tick as appropriate
  (a) Traditional rulers
  (b) Chairmen/officials of LGAs
  (c) Community religious leaders
  (d) Primary health centers
  (e) Major markets
  (f) Major motor parks
  (g) Traditional birth attendants
  (h) Schools
(17) Does your team have any nutritional programme for malnourished cleft babies?
  Yes
  No
(18) Does your team support patients with transport fare?
  Yes
  No
(19) Is your team aware of the personal qualities needed for team members?
  Yes
  No
(20) If yes, can you list five (5) of such qualities
  (a) ——————
  (b) ——————
  (c) ——————
  (d) ——————
  (e) ——————
(21) Which of the delivery methods for your awareness message have been most effective?
  (a) Radio advert
  (b) Posters
  (c) Banners
  (d) Hand bills
  (e) Souvenirs
  (f) Television advert
  (g) Newsprints
  (h) Town criers
  (i) Telephone SMS
  (j) Others, please specify
(22) Does your team recommend that mobilization and awareness activities should be handled by a separate body and are not the responsibility of the team?
  Yes
  No
(23) If yes, should the body be
  (a) Institutional Information Unit
  (b) An independent NGO
  (c) State Information Service/Ministry of Health
  (d) Federal Ministry of Health/Information
(24) What are the challenges to effective community mobilization and awareness creation for cleft services? Please list in order of importance.
  (a) ——————
  (b) ——————
  (c) ——————
  (d) ——————
  (e) ——————
